# High and Low Levels of *ABCB1* Expression Are Associated with Two Distinct Gene Signatures in Lung Tissue of Pulmonary TB Patients with High Inflammation Activity

**DOI:** 10.3390/ijms241914839

**Published:** 2023-10-02

**Authors:** Ekaterina N. Pavlova, Larisa N. Lepekha, Ekaterina Yu. Rybalkina, Ruslan V. Tarasov, Ksenia A. Sychevskaya, Elena E. Voronezhskaya, Alexander G. Masyutin, Atadzhan E. Ergeshov, Maria V. Erokhina

**Affiliations:** 1 Central Tuberculosis Research Institute, 107564 Moscow, Russia; guchia@gmail.com (E.N.P.); imber.acidis@gmail.com (A.G.M.); 2 Faculty of Biology, Lomonosov Moscow State University, 119234 Moscow, Russia; 3 FSBI N.N. Blokhin National Medical Research Center of Oncology, 115478 Moscow, Russia; 4 Koltzov Institute of Developmental Biology of Russian Academy of Sciences, 119334 Moscow, Russia; 5 Director of the Institute, Central Tuberculosis Research Institute, 2 Yauzskaya Alleya, 107564 Moscow, Russia; cniit@ctri.ru

**Keywords:** *ABCB1*, *STAT3*, pulmonary tuberculosis, chronic inflammation, signature of gene expressions

## Abstract

P-glycoprotein (encoded by the *ABCB1* gene) has a dual role in regulating inflammation and reducing chemotherapy efficacy in various diseases, but there are few studies focused on pulmonary TB patients. In this study, our objective was to identify a list of genes that correlate with high and low levels of *ABCB1* gene expression in the lungs of pulmonary TB patients with different activity of chronic granulomatous inflammation. We compared gene expression in two groups of samples (with moderate and high activity of tuberculomas) to identify their characteristic gene signatures. Gene expression levels were determined using quantitative PCR in samples of perifocal area of granulomas, which were obtained from 65 patients after surgical intervention. Subsequently, two distinct gene signatures associated with high inflammation activity were identified. The first signature demonstrated increased expression of *HIF1a*, *TGM2*, *IL6*, *SOCS3*, and *STAT3*, which correlated with high *ABCB1* expression. The second signature was characterized by high expression of *TNFa* and *CD163* and low expression of *ABCB1*. These results provide insight into various inflammatory mechanisms and association with P-gp gene expression in lung tissue of pulmonary TB patients and will be useful in the development of a host-directed therapy approach to improving the effectiveness of anti-TB treatment.

## 1. Introduction

Pulmonary tuberculosis (TB) is an infectious disease caused by *Mycobacterium tuberculosis* (*M.tb*). TB granulomas lead to chronic inflammation in the lungs, tissue damage and organ dysfunction. According to the World Health Organization, TB is one of the 10 leading causes of death in the world. Despite the success of antibiotic therapy, there has been an increase in the number of drug-resistant TB cases [1]. The proportion of TB caused by drug-resistant pathogen strains has been growing since 2014 in different regions of the world [2]. Currently, resistance of *M.tb* to one of the newest anti-TB drug bedaquiline has also been reported [3,4]. TB treatment is an expensive and time-consuming process. Therefore, there is a constant search for new targets to increase the effectiveness of TB chemotherapy. The development of host-directed therapy (HDT) is considered as a crucial direction, through which it may be possible to increase the effectiveness of anti-TB therapy and reduce inflammation to prevent subsequent damage to lung tissue [5,6,7,8]. As part of this direction, the search for prognostic biomarkers and new targets for therapeutic interventions is carried out [8,9,10]. Of particular interest in this context is P-glycoprotein (P-gp), which is able to alter the pharmacokinetics of anti-TB drugs, reducing their accumulation in the lungs and macrophages infected with *M.tb* [11].

P-gp was discovered in 1976 [12] by researchers Juliano and Ling, who named it after the capital letter of the word “Permeability”. P-gp belongs to the ATP Binding Cassette superfamily, encoded by the *ABCB1* gene, and transports compounds of various chemical nature from the cytoplasm to the extracellular environment. P-gp has wide substrate “omnivorousness”: the list of substrates for P-gp is constantly updated and includes hundreds of drugs according to DrugBank database [13]. Moreover, there is evidence suggesting that P-gp may transport endogenous substrates, possibly including cytokines [14]. The interaction between anti-TB drugs and P-gp is a relevant concern, as studies have demonstrated that rifampicin, a key anti-TB drug, is a substrate for P-gp [11,15,16,17]. High expression of P-gp has been observed in human lung cells [15] as well as in various immune cell types [16].

In different diseases, P-gp plays a critical role in both regulating inflammation activity [17,18] and reducing chemotherapy efficacy [19]. The presence of the pathogen [20], hypoxia [21,22], inflammation [23,24] and rifampicin [25,26] induce *ABCB1* gene expression in different cell types, including immune cells. These factors are relevant to TB but the underlying mechanisms behind *ABCB1* expression and P-gp function in TB remain unknown.

In this study, our objective was to identify a list of genes that correlate with high and low levels of *ABCB1* gene expression in the lungs of pulmonary TB patients with different activity of chronic granulomatous inflammation. We used samples of lung tissues obtained from patients diagnosed with pulmonary tuberculoma. In addition, all of them in the preoperative periods received anti-TB treatment the duration of which varied widely from 4 months to 5.5 years (average mean 25 months). Up to 18% of patients with pulmonary tuberculosis have this form of tuberculosis, and surgical intervention is recommended for them [27,28,29]. We compared gene expression in two groups of samples (with moderate and high activity of tuberculomas) to identify their characteristic gene signatures.

We conducted a correlation analysis between *ABCB1* expression and three groups of genes: (i) transcription factors that regulate inflammation, including *STAT 1-3-6*, *SOCS3* [30,31,32,33,34] and hypoxia-related markers *HIF1a* [35,36,37]; (ii) key cytokines involved in granuloma formation and the regulation of TB inflammation, such as *IL6*, *IL10*, *INFg*, *TGFb1*, *TNFa*, *IL1b* [38,39]; (iii) markers of pro-inflammatory (M1) and anti-inflammatory (M2) macrophages, *CD86*, *CD206*, *CD163*, *TGM,* as well as T-lymphocytes markers *CD3D*, *GZMB*.

Macrophages are key cells in controlling granulomatous inflammation during TB and serve as the primary host cells for *M.tb* [40]. The M1/M2 ratio of macrophages is considered a prognostic marker for the severity of TB [41,42]. *ABCB1* expression is higher in M2 macrophages [43]. The Human Protein Atlas project has indicated that high levels of ABCB1 expression are typical in T-lymphocytes, especially cytotoxic lymphocytes, compared to other immune cell types [44].

Additionally, analyzing the expression of transglutaminase type 2 (*TGM2*) is relevant. TGM2 has various other functions in addition to being a macrophage M2 marker [45]. A high level of TGM2 is associated with infiltration by immune cells, which has been shown using tumor tissue as an example [46,47]. It is also considered as a prognostic marker in lung cancer [48], and may be involved in the pathogenesis of TB [49].

We propose that this panel of genes will enable a comprehensive comparison of PCR analysis data with the morphological characteristics of granulomas (tuberculomas) with signs of moderate or high inflammatory activity, facilitating the identification of key genes associated with high or low levels of *ABCB1* expression in lung tissues of TB patients.

## 2. Results

### 2.1. Morphological Characteristics of Pulmonary Tuberculoma with Moderate and High Activity of Tuberculosis Inflammation

**Figure 1:**
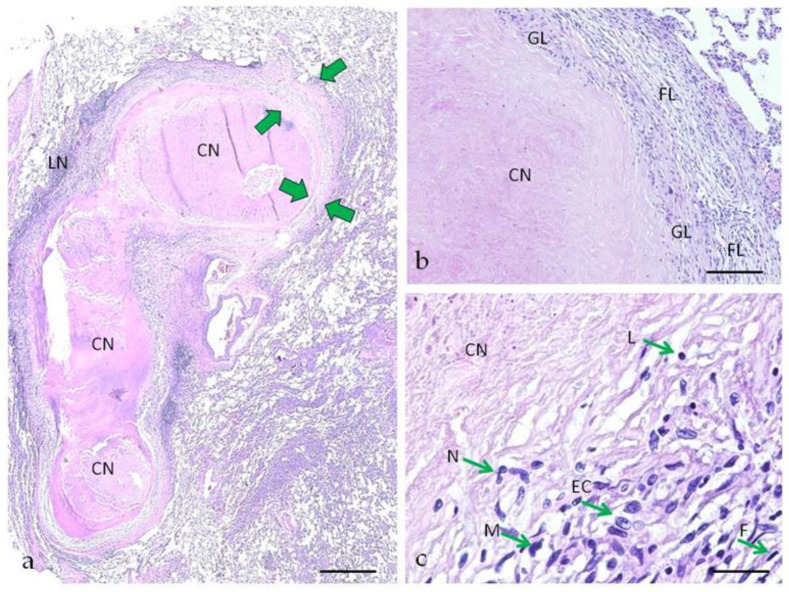
Pulmonary tuberculoma with moderate inflammatory activity of TB inflammation. (**a**)—the granuloma (conglomerate of cells) with compacted necrosis, delimited by a two-layer capsule; green arrows show the boundaries of the capsule; (**b**)—an enlarged fragment of granuloma with a fibrous layer of the capsule; (**c**)—a large fragment of the capsule wall, cells are marked with green arrows. CN—caseous necrosis, GL—granulation layer, FL—fibrous layer, LN—lymphonoduli, N—neutrophil, M—macrophage, L—lymphocyte, F—fibroblast, EC—epithelioid cell. Hematoxylin and eosin staining. Scale bars (**a**) = 500 µm, (**b**) = 100 µm, (**c**) = 20 µm.

For pulmonary tuberculoma with moderate activity of the inflammatory process, the following morphological features are characteristic: in the center of the granuloma there is an area of compacted caseous necrosis, which is surrounded by a two-layer capsule consisting of granulation (inner) and fibrous (outer) layers. In the perifocal area, the alveoli retain their normal structure (Figure 1a). The fibrous layer of the capsule prevails over the granulation layer, is well expressed, and is formed by compactly located connective tissue fibers (Figure 1b). Unlike the fibrous layer, the granulation layer is weakly expressed, represented by a few cells, among which macrophages, lymphocytes, as well as individual neutrophils and epithelioid cells are detected, single Langhans cells can also be found (Figure 1c).

**Figure 2:**
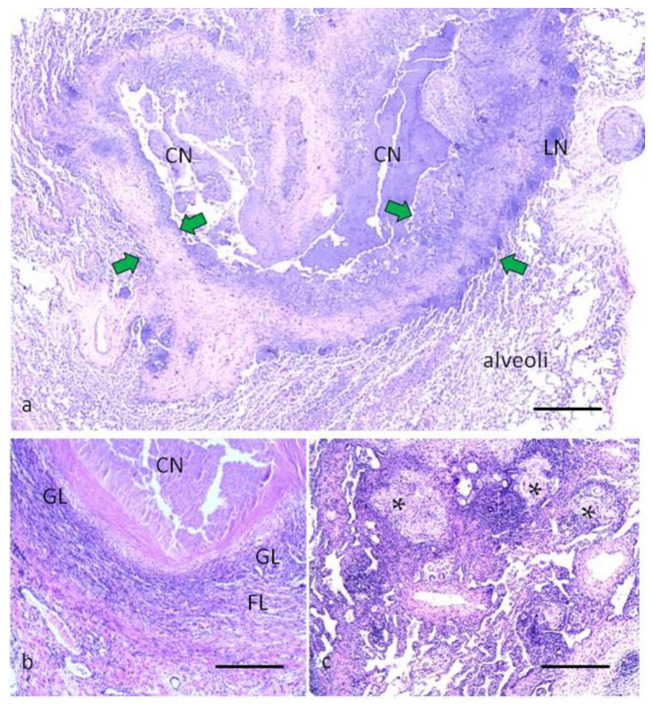
Pulmonary tuberculoma with high activity of TB inflammation. (**a**)—general view of granuloma with signs of decay of caseous necrosis; green arrows show the capsule boundaries; (**b**)—an enlarged fragment of granuloma with a granulation layer of the capsule and a developing fibrous layer; (**c**)—dropout centers. CN—caseous necrosis, GL—granulation layer, FL—fibrous layer, LN—lymphonoduli, *—zones of the spread of tuberculosis infection. Hematoxylin and eosin staining. Scale bars (**a**) = 500 µm, (**b**,**c**) = 100 µm.

Tuberculomas with a high activity of the inflammatory process are characterized by caseous necrosis with signs of decay, a predominance of the granulation layer and a weakly expressed fibrous layer of the capsule (Figure 2). In the granulation layer, inflammatory cells, including neutrophils, are determined. The formation of lymph nodes along the periphery of the capsule is observed. The fibrous layer is poorly expressed and is in the process of formation. In the perifocal area, “screening centers” are determined—lympho-hematogenous zones of the spread of tuberculosis infection.

As a result of the morphological analysis, 13 patients from 2017 to 2018 and 15 patients from 2021 to 2022 were selected in the group of tuberculomas with signs of a moderate inflammatory process, in the group of tuberculomas with signs of a high inflammatory process—17 and 20 patients, respectively.

### 2.2. Expression of ABC-Transporter (P-gp, MRP1, BCRP) Genes in Lung Tissue of TB Patients with Pulmonary Tuberculoma

A study conducted on the samples obtained in 2017–2018 used real-time PCR to determine the expression of genes coding for ABC drug transporter proteins, including P-glycoprotein (P-gp, *ABCB1*), Multidrug Resistance associated protein (MRP1, *ABCC1*), and Breast cancer resistance protein (BCRP, *ABCG2*), in the surgical material of lungs of patients diagnosed with pulmonary tuberculoma (n = 30).

**Figure 3:**
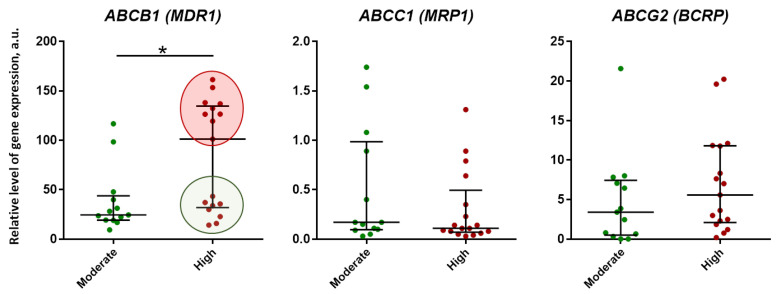
Relative level of gene expression of ABC transporter proteins depending on the activity of inflammation (Moderate and High) in the surgical material of patients diagnosed with multiple pulmonary tuberculomas. According to the level of *ABCB1* gene expression in the group with high inflammation activity, two subgroups are distinguished: with high expression (figure with red fill) and low expression (green fill). Data are presented as median and interquartile range (25% and 75%). The relative level of gene expression was calculated as 2^−ΔCt^ × 10^3^ and expressed in arbitrary units (a.u.). Statistical analysis was performed using the Mann–Whitney U-test. Significant differences between groups: *—*p* < 0.05.

The study found significant variability in the expression of these genes, with high values of relative expression level typical for *ABCB1*—36.34 (23.02–118.81) a.u., medium values for *ABCG2*—3.75 (1.36–7.96) a.u, and low values for *ABCC1*—0.14 (0.08–0.58) a.u. The analysis also showed that only *ABCB1* gene expression was significantly higher (*p* = 0.02) in samples with high inflammation activity compared to moderate activity. In the group with high inflammation activity, two subgroups with different levels of *ABCB1* expression were identified: a subgroup with high expression, significantly higher than the values in the group with moderate inflammation activity (figure with red fill), and a subgroup with low expression (figure with green fill), matching the values in the moderate group (Figure 3).

These findings suggest that the expression of ABCB1 at high inflammation activity is heterogeneous and can be influenced by various factors. This hypothesis was tested on samples collected during the period 2021–2022, and the results are presented below.

### 2.3. Correlation Analysis of Genes Expression in Lung Tissue of TB Patients

To investigate the relationship between *ABCB1* expression and inflammatory activity, we analyzed the correlation between *ABCB1* and various genes, including cytokines (*IL1*, *IL6*, *IL10*, *TNFa*, *TGFb1,* and *INFg*), transcription factors (*STAT1-3-6*, *SOCS3*, and *HIF1a*), and markers of macrophages (*CD86*, *CD163*, *CD206*, and *TGM2*) and T-lymphocytes (*CD3D* and *GZMB*).

**Figure 4:**
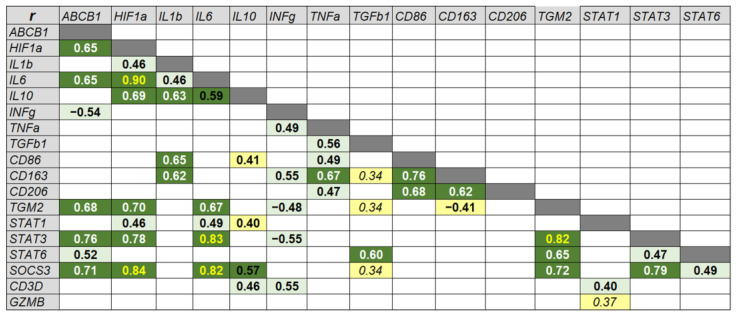
The matrix of significant nonparametric Spearman’s coefficients of correlation (r) between the levels of gene expression. Color of the cells indicates significant differences: *p* < 0.05—yellow, *p* < 0.01—green, *p* < 0.001—dark green. The value of coefficient r = 0.2–0.39 as weak (black italic), 0.40–0.59 as moderate (black bold), 0.6–0.79 (white bold) as strong and 0.8–1 (yellow bold) as very strong correlation.

We found that *ABCB1* has the highest correlation with *STAT3* and *SOCS3* (r = 0.76 and 0.71, respectively) (Figure 4).

Additionally, *ABCB1* expression was strongly correlated with *TGM2* (r = 0.69), *HIF1a* (r = 0.65) and *IL6* (r = 0.65) genes, while moderately positive correlated with *STAT6* (r = 0.52) and negatively correlated with *INFg* (r = −0.54).

**Figure 5:**
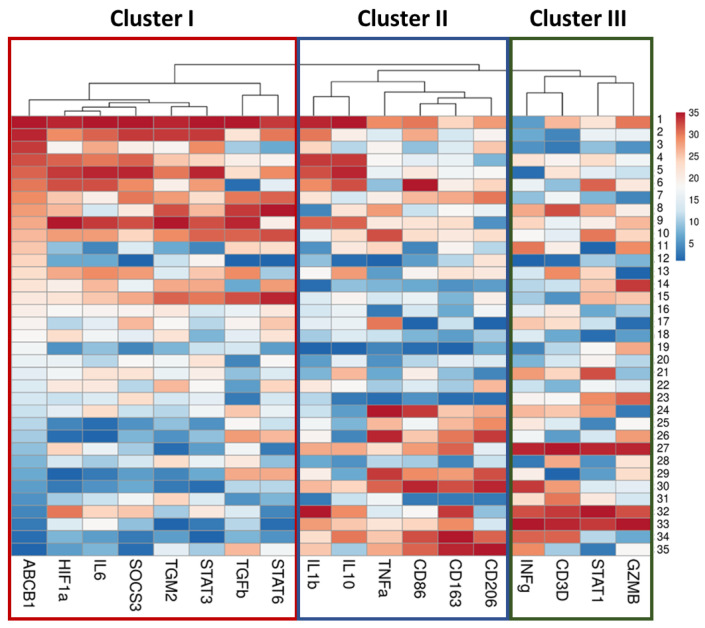
Heatmap correlation of co-expression genes in the lung tissue of patients with pulmonary granulomas reveals 3 clusters. Columns are clustered using correlation distance and average linkage. Expression of the *ABCB1* gene is ranked in descending order. A three-color scale was used with blue indicating low, white—average, and red representing high relative level of gene expression. This heatmap was performed on rank-transformed relative gene expression using the webtool ClustVis (https://biit.cs.ut.ee/clustvis/, accessed on 25 June 2023).

Using hierarchical cluster analysis based on correlation, we identify three large clusters of co-expressed genes (Figure 5).

Cluster I, including *ABCB1* gene, contains predominant transcription factor genes such as *STAT3*, *SOCS3*, *HIF1a*, cytokine *IL6* and *TGM2*. *IL6* gene expression demonstrates a very strong correlation with *HIF1a* (r = 0.91), *STAT3* (r = 0.83) and *SOCS3* (r = 0.82). In turn, *STAT3* expression strongly correlates with *SOCS3* expression (r = 0.79). *TGM2* gene expression exhibits a very strong correlation with *STAT3* (r = 0.82) and a strong correlation with *SOCS3* (r = 0.72). *HIF1a*, in addition to the genes listed above, strongly correlates with *STAT3* (r = 0.78) and *IL10* (r = 0.69). Also in this cluster, a separate subcluster of *STAT6* and *TGFb1* is distinguished, which most closely correlate with each other (r = 0.601). At the same time, *STAT6* has a strong correlation with *TGM2* (r = 0.65) and moderate correlation with *ABCB1* (r = 0.52) and *STAT3* (r = 0.47).

Cluster II includes predominantly macrophage markers CD86, CD206, and CD163, which correlate most closely with *TNFa* gene expression. Interestingly, the expression of *CD163* gene, which is predominantly a macrophage M2 marker, correlates to a greater extent with *CD86*, a macrophage M1 marker (r = 0.77), than with the expression of another M2 marker, *CD206* (r = 0.62). *CD163* also has a strong correlation with *TNFa* (r = 0.67) and *IL1b* (r = 0.62). However, no significant correlation was found between the expression of *IL1b* and *TNF-α*. In this cluster, *IL1b* and *IL10* form a separate subcluster, the expression of which strongly correlates with each other (r = 0.63).

Cluster III is represented mainly by genes of T-lymphocytes markers. The expression of the gene marker of cytotoxic lymphocytes *GZMB* is combined into one subcluster with *STAT1*, despite the fact that a weak correlation was found between them (r = 0.37), and *CD3D* with *INFg* moderately correlate with each other (r = 0.55). It should be noted that the expression of *INFg* gene included in this cluster, in addition to *ABCB1* gene, also exhibits moderate negative correlations with *STAT3* (r = −0.55) and *TGM2* (r = −0.49).

Clusters II and III are hierarchically related to each other, but not to Cluster I.

### 2.4. Analysis of Genes Expression in Lung Tissue of TB Patients Depending on the Activity of Inflammation

**Figure 6:**
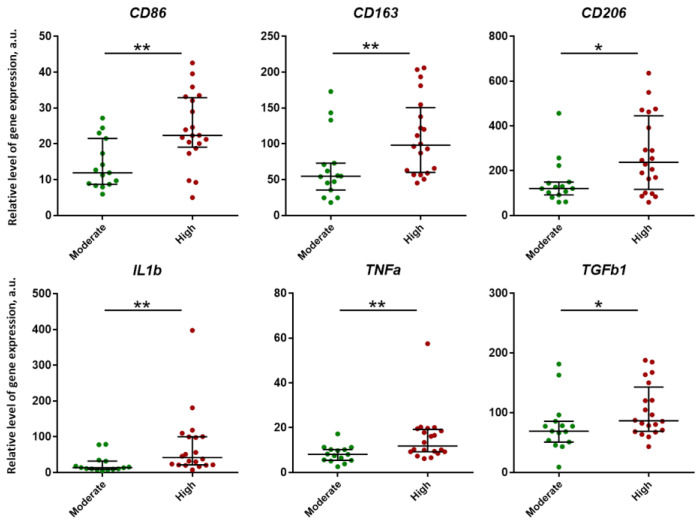
Significant differences in the level of expression between pulmonary granulomas with moderate and high inflammation activity. Data are presented as median and interquartile range (25% and 75%). The relative level of gene expression was calculated as 2^−ΔCt^ × 10^4^ and expressed in arbitrary units (a.u.). Statistical analysis was performed using Mann–Whitney U-test. Asterisks indicate significant differences: *, *p* < 0.05; **, *p* < 0.01.

The relative level of expression of cytokine genes such as *IL1b*, *TGFb1* and *TNFa*, and macrophage markers *CD86*, *CD163* and *CD206* was higher in the lung tissue of patients with high inflammation activity (“High” group) compared to moderate (“Moderate” group) (Figure 6). No statistically significant differences were found in the level of expression of all other studied genes.

**Figure 7:**
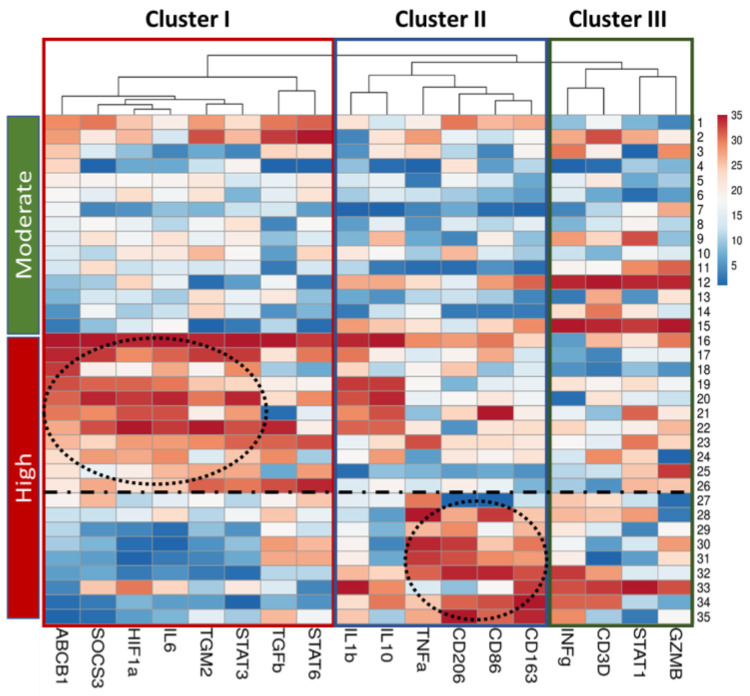
The heatmap demonstrates the heterogeneity of the genetic signature in a group with high inflammation activity. The relative level of *ABCB1* gene expression is ranked in descending order in each group with moderate (Moderate) and high (High) inflammatory activity. A three-color scale was used, where blue indicated low, white—medium and red—high relative level of gene expression. This heatmap was performed on rank-transformed relative gene expression using the webtool ClustVis (https://biit.cs.ut.ee/clustvis/, accessed on 25 June 2023).

According to the heatmap data presented, the “High” group exhibits heterogeneity in gene expression levels. Within this group, some samples are predominantly characterized by high expression of genes in Cluster I, while others show predominantly high expression in Cluster II (Figure 7).

Based on the gene expression profile, primarily by the level of *ABCB1* expression, we divided the group with high activity inflammation into two subgroups: the first subgroup included samples with line numbers 16–26 (n = 11), the second subgroup included samples with line numbers 27–35 (n = 9) (Figure 7). We conducted a comparative gene expression analysis in these subgroups against “Moderate” group for each of the identified clusters.

**Figure 8:**
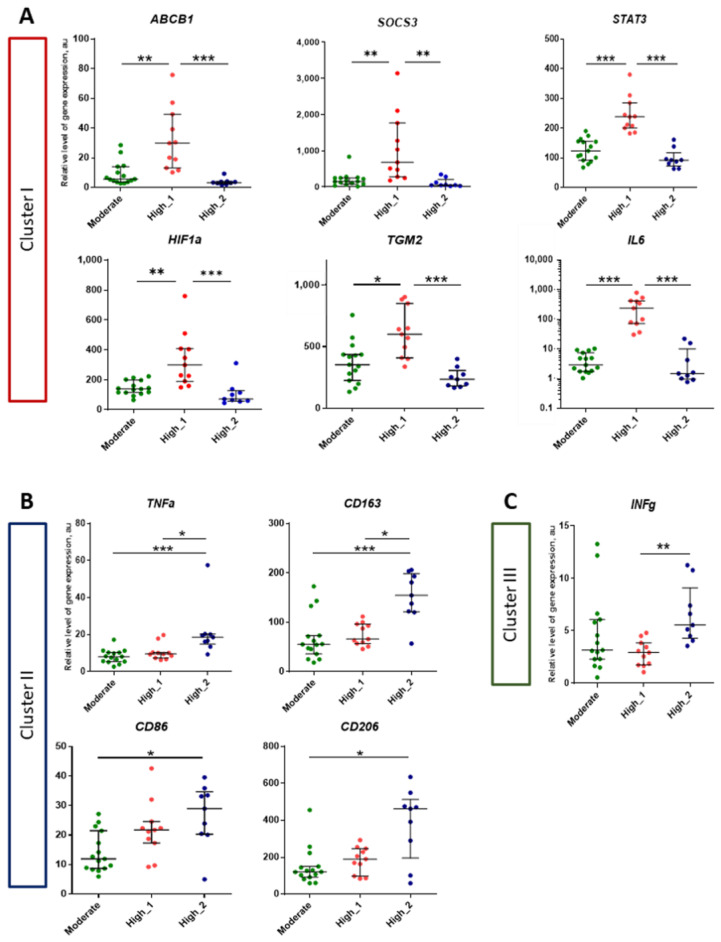
Analysis of gene expression in the lung tissue of patients with different activity of TB inflammation in Clusters I–III (**A**–**C**). Data are presented as median and interquartile range (25% and 75%). The relative level of gene expression was calculated as 2^−ΔCt^ × 10^4^ and expressed in arbitrary units (a.u.). Statistical analysis was performed using Kruskal–Wallis’s test followed by the Dunn’s multiple comparison test. Asterisks in the graphs indicate significant differences: *, *p* < 0.05; **, *p* < 0.01; ***, *p* < 0.001.

A comparative analysis of gene expression showed that in Cluster I, the expression of *ABCB1*, *IL6*, *SOCS3*, *STAT3*, *HIF1a*, and *TGM2* in “High-1” subgroup is higher than in “High-2” subgroup. The median relative level of expression of these genes in “High-1” subgroup is also significantly higher than in “Moderate” group. At the same time, the expression of these genes in “High-2” subgroup does not differ from “Moderate” group (Figure 8A).

In Cluster II, the expression of *TNFa* and *CD163* genes in “High-2” subgroup is higher than in “High-1” subgroup and the median expression of these genes in “High-2” subgroup is significantly higher than in “Moderate” group, while the expression of these genes in “High-1” subgroup does not differ from “Moderate”. The expression of *CD86* and *CD206* genes in “High-2” subgroup significantly exceeds the values in “Moderate” group. However, we did not find significant differences in the expression of these genes between the “High-1” and “High-2” subgroups (Figure 8B).

In Cluster III, differences were found only in the level of *INFg* gene expression between the “High-1” and “High-2” subgroups. At the same time, no difference was found between these two subgroups with the “Moderate” group (Figure 8C).

**Figure 9:**
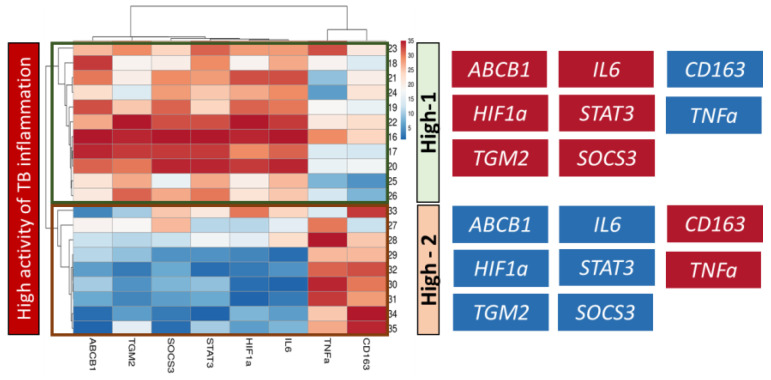
Genetic signatures of gene expression characteristic of each of the subgroups with high inflammation activity in the lung tissue of TB patients. A three-color scale was used with blue indicating low, white—average, and red representing high relative level of gene expression. This heatmap was performed on rank-transformed relative gene expression using the webtool ClustVis (https://biit.cs.ut.ee/clustvis/, accessed on 25 June 2023).

Thus, differences in the level of gene expression between subgroups with high inflammation activity and their comparison with the group with moderate inflammation activity made it possible to identify two different genetic signatures that may cause high inflammation activity. The first subgroup of genes includes *ABCB1*, *HIF1a*, *SOCS3*, *STAT3*, *IL6* and *TGM2*, and the second subgroup of genes includes *CD163* and *TNFa*. At high inflammation activity, as analysis shows, either one or the other signature predominates in the lung tissue of TB patients (Figure 9).

## 3. Discussion

In this study, we utilized surgically acquired samples exclusively from patients diagnosed with pulmonary tuberculoma. Focusing only on this clinical form allowed us to standardize the samples for analysis and eliminate the influence of factors associated with other forms of TB. We identified differences in *ABCB1* gene expression and its correlation with other genes depending on the inflammation activity of tuberculomas.

On the samples obtained in 2017–2018, we investigated the expression of the most clinically relevant ABC drug transporter genes, such as *ABCB1* (P-gp), *ABCC1* (MRP1), and *ABCG2* (BCRP), expressed in the lungs [50]. Our findings revealed higher expression of the ABCB1 gene in patients with active inflammation activity. The same result was confirmed during the PCR analysis of samples obtained in 2021–2022. Notably, both materials also revealed the heterogeneity of *ABCB1* gene expression in the lung tissue with a high activity of inflammation in lung tissue. This confirms that high activity of inflammatory process (according to the morphological assessment of pulmonary tuberculoma) can be accompanied by both high and low expression of *ABCB1* gene. This raises the question of identifying genetic profile differences between subgroups with varying levels of *ABCB1* expression.

It is assumed that the functional activity of P-gp and polymorphisms in *ABCB1* gene may reduce the accumulation of anti-TB drugs in the foci of TB inflammation [51,52]. Consequently, this can lead to the selection/emergence of drug-resistant strains of *M.tb* [11], and ultimately contribute to inflammation progression. Thus, our goal was to explore the relationship between various genes associated with high inflammation activity and *ABCB1* gene expression in the lung tissue of TB patients. Various factors involved in inflammation during TB can act as inducers of *ABCB1* gene expression.

Cytokines such as IL1b, IL6, TNFa, INFg, and TGFb1 have been shown to increase the expression of *ABCB1* [23,24]. Transcription factors NF-kB and STAT3, which play crucial roles in regulating inflammation in TB, directly bind to the promoter region of *ABCB1* gene and induce its expression [53,54,55]. Human TB lesions are severely hypoxic and *M.tb* infection drove HIF1α accumulation even in normoxia [36]. Notably, HIF1a, a transcription factor involved in hypoxia responses, was found to have a binding site within *ABCB1* gene [56].

TB is characterized by the formation of granulomas, organized aggregates of immune cells. Cellular composition and gene expression analyses within granulomas during TB progression have received considerable attention [57,58,59,60]. It is important to note that individual lung granulomas within the same patient exhibit heterogeneity in terms of inflammation activity, cellular composition, and the ability of their cells to eliminate *M.tb* [61]. In our study, we analyzed gene expression not in the granuloma itself, but in the area of the lung tissue adjacent to the wall of the granuloma, so-called perifocal area (PFA). Lung tissues surrounding TB foci are sensitized and highly responsive to tuberculin, even when injected at a distant site [62]. We hypothesized that gene expression in the PFA would reflect the systemic nature of inflammation and immune system parameters to a greater extent than directly within the granulomas.

In TB inflammation, the cellular composition of PFA changes: CD3+ and CD68+ cells are detected, although in low levels compared to granuloma [57]. We previously demonstrated the presence of *M.tb* aggregates and clusters within the PFA [63]. At high inflammation activity, we also observed PFA infiltration by inflammatory cells and the formation of granulomas without foci of necrosis. We hypothesized that these infiltrates may include immune cells with high expression of *ABCB1*.

ABCB1 expression is characteristic of many types of immune cells [16]. Although we have not found a correlation between *ABCB1* expression and lymphocyte or macrophage markers in this study, we believe that further analysis of lung tissue in TB patients using multiplex transcriptomics (e.g., single-cell RNA sequencing) or proteomics is required to identify specific cell types associated with high *ABCB1* gene expression.

We observed that, overall, the expression levels of macrophage markers *CD86, CD163*, and *CD206* were higher in the group with high inflammation activity compared to the moderate group. Suzuki et al. found that the expression levels of CD206 and CD163 proteins in lung tissue correlated with their soluble forms in the blood serum of TB patients. Furthermore, plasma concentrations of sCD206 and sCD163 were higher in patients with active pulmonary TB compared to control subjects, and increased levels of sCD163 and sCD206 were associated with higher mortality in pulmonary TB patients [64,65]. Serum sCD163 concentrations also correlated with TB severity [66]. 

We observed the highest expression of *CD163* gene in the group of patients characterized by low expression of the *ABCB1* gene and high expression of *TNFa* gene. Additionally, in the high inflammation activity group, the expression levels of *TNFa*, *IL1b*, and *TGFb1* cytokine genes were generally higher compared to the moderate inflammation activity group. Numerous studies have reported that elevated levels of these cytokines correlate with TB severity. Serum levels of TNFa, IL1b, and TGFb1 were found to be related to radiological severity in patients with active pulmonary tuberculosis [67]. Similarly, serum levels of TNFa and TGFb were significantly higher in the advanced TB cases when compared with mild–moderate TB patients [68]. Patients with pulmonary TB and bilateral or cavitary lesions of lung tissue had significantly higher levels of TNFa and IL1b compared with those with unilateral or non-cavitary lesions [69]. Serum TNFa levels correlate with the increase in clinical severity score in TB patients [70,71]. According to our data, TNFa expression demonstrated a strong correlation with CD163 expression. Moreover, the macrophage genes were grouped together in a gene co-expression cluster with TNFa.

Furthermore, while *TGFb1* moderately correlated with *TNFa* expression (r = 0.56), we identified a strong correlation between *TGFb1* expression and *STAT6* (r = 0.60), with *STAT6* moderately correlating with *STAT3* (r = 0.47). Protective immunity against *M.tb* is believed to require activation of Type 1 INFg-dependent signaling pathway [72]. However, it has been shown that both Type 1 and Type 2 signaling pathways may be required for mycobacterial granuloma formation [32]. Therefore, the increased level of *TGFb1* expression in the lung tissue of TB patients with high activity inflammation may be associated with the predominance of Type 2 signaling pathways. In our correlation analysis, we found a negative moderate correlation between *STAT3* and *INFg* gene expression.

TNFa, a pro-inflammatory cytokine, plays a crucial role in the immune response against *M.tb* [73]. It is produced by various immune cells, particularly macrophages, in response to mycobacterial infection and is required for pathogen control. TNFa signaling activates the canonical NF-κB pathway. However, excessive and uncontrolled production of TNFa can lead to tissue damage and hyperinflammation [74]. Interestingly, Beig et al. found that some patients had normal serum TNFa values despite severe lymphadenopathy and necrosis, suggesting the involvement of factors other than TNFa in high inflammation activity [71].

We found that two subgroups, “High-1” and “High-2”, are distinguished at high inflammatory TB activity. “High-1” is characterized by high levels of gene expression, indicated in the heatmap as Cluster I, and “High-2” is characterized by a high level of gene expression from Cluster II. Analyzing gene expressions between these clusters allowed us to identify two genetic signatures characterizing these subgroups. Importantly, either one or the other signature predominated in cases of high inflammation activity. Notably, no significant morphological changes were observed between these subgroups on standard H&E histological sections. It is possible that further analysis of cellular composition using additional cellular markers and immunohistochemistry methods could reveal such differences.

We previously mentioned *TNFa* and *CD163*, as their expression was generally higher in the group with high inflammation activity. In the other subgroup, the median expression of *ABCB1*, as well as *IL6*, *STAT3*, *SOCS3*, *HIF1a*, and *TGM2*, was significantly higher compared to the group with moderate inflammation activity.

IL6, similar to TNFa, plays a role in the immune response against *M.tb* by activating immune cells and promoting the production of other pro-inflammatory cytokines. Elevated plasma levels of IL6 have been observed in TB patients with more severe disease and are associated with pulmonary inflammation and lung damage [75,76]. IL-6 signaling primarily occurs through JAK/STAT pathway, predominantly via STAT3 [77]. High levels of IL6, like TNF-a, can result in tissue damage and hyperinflammation [78].

**Figure 10:**
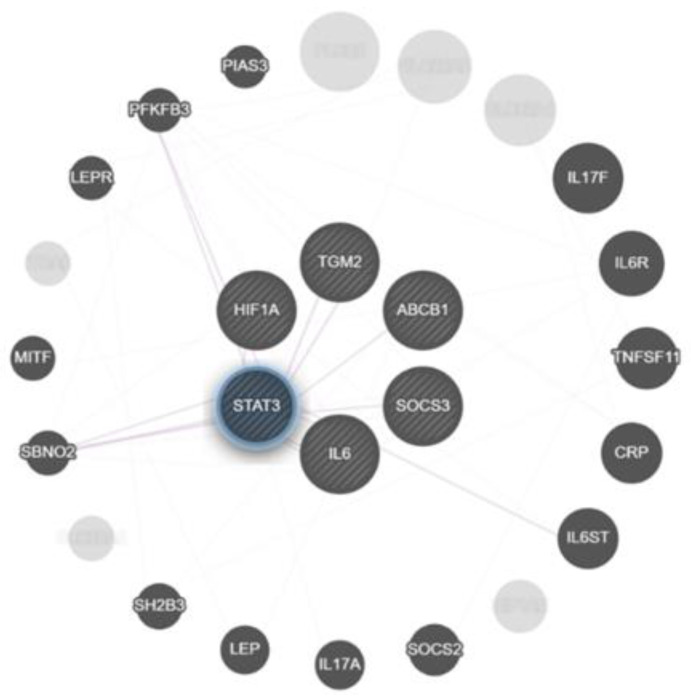
Interaction network between genes based on their co-expression as retrieved by GeneMANIA. Two genes are linked if their expression levels are similar across conditions in a gene expression study.

Utilizing the GeneMANIA.org resource, we constructed a network of interactions between genes, which not only correlated with the expression of *ABCB1*, but were also increased at high inflammation activity. *STAT3* was found to represent a core gene that connects all these genes (Figure 10).

It is known that STAT3 plays an important role in the pathogenesis of TB [30]. Single nucleotide polymorphisms of the STAT3 gene are associated with vulnerability to active TB or correlate with disease severity [79].

STAT3 (Signal Transducers and Activators of Transcription) is a component of the IL6-activated Acute Response Phase Factor (APRF) complex that stimulates the production of innate immune mediators [80]. In the cell, it can be in un-phosphorylated (uSTAT3) and phosphorylated (pSTAT3) form. IL6 stimulates the formation of pSTAT3, which activates many genes, including the STAT3 gene itself. This results in an increase in the concentration of uSTAT3 which drives a second wave of expression of IL6 [81]. The SOCS3 (cytokine signaling suppressor 3) inhibits STAT3 phosphorylation by binding to the gp130 subunit of IL6 receptor. While SOCS3 is generally a negative regulator of IL6 signaling, it can positively regulate inflammatory responses by inhibiting STAT3 in certain cases. Aberrant expression levels of SOCS3/STAT3 have been reported in various cell types, suggesting their involvement in the pathogenesis of various inflammatory diseases [82].

IL6, IL10, pSTAT3, and SOCS3 have been identified as influential factors distinguishing tuberculosis patients from healthy individuals [83]. Although *IL10* was not included in the same cluster as *IL6* gene expression in our study, we found moderate correlations between *IL10* expression and *IL6* (r = 0.59) and *SOCS3* (r = 0.57). Previous studies have demonstrated a correlation between high serum levels of IL6 and IL10 in the blood of patients with active TB, which has been attributed to aberrant activation of STAT3 in immune cells [84].

Initially, we included TGM2 in our study as a macrophage M2 marker. We observed a negative moderate correlation between *TGM2* and *CD163* expression (r = −0.41). TGM2 is a pleiotropic enzyme involved in various biological functions, including post-translational protein modifications, cell death, and signaling [85]. It has been shown that TGM2 activates the IL-6/JAK/STAT3 signaling pathway, and suppressing TGM2 expression reduces STAT3 phosphorylation [86]. In TB inflammation, TGM2 plays a significant role, promoting the survival of *M.tb* in infected macrophages: its inactivation enhances the bactericidal properties of macrophages. TGM2 may be a potential target for HDT for TB [87]. In addition, TGM2 constitutively activates the HIF1a promoter [88].

Hypoxia is a characteristic feature of TB inflammation in the lungs of patients [36]. Hypoxia-inducible factor 1 (HIF1) is a transcription factor composed of HIF1a and HIF1b subunits. While HIF1b is constitutively expressed, HIF1a levels can vary. HIF1a can be stabilized by STAT3 and IL-6 [89]. In turn, STAT3 inhibition blocks HIF1a expression [90]. The role of HIF1a in tuberculosis is ambiguous: it promotes antimicrobial activity of human macrophages against *M.tb*, but also impairs CD4 T cell activation and differentiation, leading to increased susceptibility to *M.tb* infection [91]. HIF1a restricts NF-κB-dependent gene expression to control innate immunity signals, thereby preventing excessive and damaging pro-inflammatory responses [92]. Hence, high HIF1a expression can contribute to pathogen destruction, but its excessive activation can also lead to inflammation progression and tissue damage [93]. Comparing our correlation analysis results with existing knowledge, we can support the relationship between high *ABCB1* expression and the expression of *IL6*, *STAT3*, *SOCS3*, *HIF1a*, and *TGM2* genes. Moreover, the correlations we identified suggest that the induction of *ABCB1* expression in the lung tissue of TB patients is mainly mediated by the activation of the STAT3 signaling pathway.

It is worth noting that elevated levels of secreted P-gp have been associated with disease severity or therapeutic response failure in conditions such as chronic rhinosinusitis, rheumatoid arthritis, and systemic lupus erythematosus [94,95,96]. *ABCB1* is included in a list of 20 differentially expressed genes in whole blood that distinguish patients with active tuberculosis from healthy individuals [97]. Increased expression and functional activity of P-gp have been found in memory CD4 T cells of patients with latent TB compared to non-infected controls [98]. These findings underscore the importance of studying the regulation of *ABCB1* gene expression and the functional activity of P-gp in patients with pulmonary TB.

## 4. Materials and Methods

### 4.1. Patients and Lung Tissue Samples

This study exclusively enrolled patients diagnosed with pulmonary tuberculoma, a clinical–anatomical form of tuberculosis (TB) according to the Russian classification system [99] and were referred for surgical treatment. The diagnosis of TB was confirmed through bacteriological and PCR methods.

Lung tissue samples were obtained from 30 patients (18 women and 12 men) aged 28–49 years (mean 35.24) in 2017–2018 and from 35 patients (14 women and 21 men) aged 18–56 years old (mean 32.43) in 2021–2022 in Central Tuberculosis Research Institute (CTRI, Moscow, Russian Federation). All patients in the preoperative periods received anti-TB treatment and its duration varied widely: from 11 months to 4 years in patients in 2017–2018 (mean 28.3 months) and from 4 months to 5.5 years in patients in 2021–2022 (mean 22.2 months).

### 4.2. Histology

Following surgical resection, lung tissue specimens were immediately fixed in 10% formalin (BioVitrum, Moscow, Russia). Dehydration was carried out according to the standard protocol in a series of alcohols from 70 to 96%, 100% ethanol was replaced by isopropyl alcohol. After dehydration with isopropyl alcohol, the samples were embedded in paraffin Hystomix (BioVitrum, Moscow, Russia), and then were cut at 5 μm thickness for preparation of serial section slides. Tissue sections were stained with hematoxylin & eosin (H&E) and embedded in Canada balsam for histological examination. Slides were used to classify the clinical material into tuberculoma with high and moderate activity of TB inflammation. A Biozero BZ-9000E from Keyence (Keyence, Osaka, Japan) was used for light microscopy. The research was carried out using the equipment of the Core Centrum of the Institute of Developmental Biology RAS.

### 4.3. Gene Expression Analyses

A sample of lung tissue (about 25–50 mg) was taken immediately after surgery from an area about 0.5 cm from the granuloma wall, referred to as the perifocal area (PFA). The samples were frozen and stored at −80 °C for subsequent PCR analysis.

RNA extraction was performed using TRI Reagent (MRC, Cincinnati, OH, USA) according to the manufacturer’s instructions. The RNA concentration was measured using a NanoDrop 8000 spectrophotometer (Thermo Fisher Scientific, Waltham, MA, USA). RNA integrity was assessed by gel electrophoresis, and samples with visible bands of 5S and 18S RNA were taken for analysis.

For cDNA synthesis, 1 μg of RNA was treated with DNase (Thermo Fisher Scientific, Waltham, MA, USA) as per the manufacturer’s instructions. Reverse transcription was performed using the MMLV RT kit (Evrogen, Moscow, Russia) following the provided instructions and utilizing random hexamer primers. The resulting cDNA was used for quantitative PCR analysis. For real-time PCR, the commercial mixture qPCRmix-HS SYBR and Low-Rox (Evrogen, Moscow, Russia) containing polymerase, buffer, and a mixture of nucleotides was used according to the manufacturer’s manual. A 250 ng sample of the synthesized cDNA was taken into the reaction, followed by the addition of a forward and reverse primer at a final concentration of 1 µM.

Real-time PCR for samples obtained between 2017 and 2018 was performed in 2018 using a CFX96 Touch (Bio-Rad Laboratories, Inc., Hercules, CA, USA) with the primers listed in Table 1.

Each sample was amplified in triplicate using the following PCR cycling profile: 95 °C for 5 min, followed by 39 cycles at 95 °C for 13 s, 60 °C for 20 s and 72 °C for 15 s. Relative expression levels were quantified using the ΔCt method and arbitrary units (a.u.) were calculated as 2^−ΔCt^ × 10^3^, where ΔCt is the difference between the threshold cycles of the target gene and the housekeeping gene RPL27.

Real-time PCR for samples obtained between 2021 and 2022 was performed in 2022 using a QuantStudio 12K Flex system (Applied Biosystems, Waltham, MA, USA) using the primers shown in Table 2. 

Each sample was amplified in duplicate using the following PCR cycling profile: 95 °C for 3 min, followed by 40 cycles at 95 °C for 15 s and 60 °C for 60 s. Relative expression levels were quantified using the ΔCt method and arbitrary units (a.u.) were calculated as 2^−ΔCt^ × 10^4^, where ΔCt is the difference between the threshold cycles of the target gene and the B2M housekeeping gene.

### 4.4. Data Analyses

Statistical processing was carried out using the statistical package GraphPad Prism Version 7.04 (GraphPad Software, La Jolla, CA, USA). The data are presented as medians with interquartile ranges. Nonparametric Mann–Whitney U-test was used for two-group comparisons, and nonparametric Kruskal–Wallis’s test followed by the Dunn’s multiple comparison test were employed. All *p*-values were two-tailed, and *p* < 0.05 was considered statistically significant. The correlation between variables was assessed using the Spearman correlation coefficient. Hierarchical clustering analysis and heatmap visualization of rank-transformed relative gene expression were performed with ClustVis 2.0 using correlation distance and average linkage (https://biit.cs.ut.ee/clustvis, accessed on 25 June 2023) [100].

## 5. Conclusions

During the analysis of gene expression in the lung tissue of TB patients, we observed that despite similar morphological characteristic of high inflammation activity, the genetic profile of the samples differed. This study provides novel evidence that high inflammation activity can arise from various factors. This can be either the development of a pathological process caused by high levels of *IL6* and *STAT3*, or a high level of *TNFa* and, possibly, infiltration of the affected area with CD163-positive macrophages. We identified a gene expression signature characterized by increased expression of *HIF1a*, *TGM2*, *IL6*, *SOCS3*, and *STAT3* genes, which not only associated with high inflammation activity but also correlated with *ABCB1* expression. Consequently, we propose that elevated *ABCB1* expression in lung tissue of TB patients is potentially mediated through the activation of the STAT3 signaling pathway. Another gene expression signature associated with high inflammatory activity was marked by elevated levels of *TNFa* and *CD163*. Moving forward, these identified signatures may improve our understanding of immunopathology regarding TB and potentially be useful in the development of an HDT approach to improving the effectiveness of anti-TB treatment and preventing lung tissue damage mediated by high inflammation activity.

## Figures and Tables

**Table 1:** Primer pairs used for real-time PCR for surgical material obtained in 2017 and 2018.

Gene	Forward Primer	Reverse Primer	Product Length, bp
*ABCB1*	GGGATGGTCAGTGTTGATGGA	GCTATCGTGGTGGCAAACAATA	110
*ABCC1*	GTGAATCGTGGCATCGACATA	GCTTGGGACGGAAGGGAATC	184
*ABCG2*	TGAGCCTACAACTGGCTTAGA	CCCTGCTTAGACATCCTTTTCAG	75
*RPL27*	ACCGCTACCCCCGCAAAGTG	CCCGTCGGGCCTTGCGTTTA	198

**Table 2:** Primer pairs used for real-time PCR for surgical material obtained in 2021 and 2022.

Gene	Forward Primer	Reverse Primer	Product Length, bp
*B2M*	GGGTTTCATCCATCCGACATTG	ACACGGCAGGCATACTC	161
*RPL27*	ACCGCTACCCCCGCAAAGTG	CCCGTCGGGCCTTGCGTTTA	198
*ABCB1*	TTGCTGCTTACATTCAGGTTTCA	AGCCTATCTCCTGTCGCATTA	105
*HIF1a*	CTGAACGTCGAAAAGAAAAGTC	AAATCACCAGCATCCAGAAGT	190
*IL1b*	TTACAGTGGCAATGAGGATGAC	TGTAGTGGTGGTCGGAGATTC	131
*IL6*	ACTCACCTCTTCAGAACGAATTG	CCATCTTTGGAAGGTTCAGGTTG	149
*IL10*	CGCTGTCATCGATTTCTTCCC	AGAGTCGCCACCCTGATGTC	185
*TNFa*	TCAGCAAGGACAGCAGAGGA	GTCAGTATGTGAGAGGAAGAGAACC	128
*INFg*	TCGGTAACTGACTTGAATGTCCA	TCGCTTCCCTGTTTTAGCTGC	93
*TGFb1*	AACAATTCCTGGCGATACCTCA	AAGCCCTCAATTTCCCCTCC	125
*STAT1*	TGGTTCACTATAGTTGCGGAGAG	TGAATGAGCTGCTGGAAAAGA	158
*STAT3*	CATATGCGGCCAGCAAAGAA	ATACCTGCTCTGAAGAAACT	152
*STAT6*	AGGATGGCAGGGGTTATGT	GGGGGATGGAGTGAGAGTG	198
*SOCS3*	AAGATCCCCCTGGTGTTGA	TTAAAGCGGGGCATCGTA	162
*CD3D*	GAGGGAACGGTGGGAACA	CAAAGCAAGGAGCAGAGTGG	228
*GZMB*	CCCTGGGAAAACACTCACAC	CCCCACGCACAACTCAAT	116
*CD86*	CAAAACCAAAGCCTGAGTGA	GGCTTTTTGTGATGGATGATAC	215
*CD163*	ATCCCGTCAGTCATCCTTTATT	CTGTCTCTGTCTTCGCTTTTTAGT	103
*CD206*	CTACAAGGGATCGGGTTTATGGA	TTGGCATTGCCTAGTAGCGTA	105
*TGM2*	CGTGACCAACTACAACTCGG	CATCCACGACTCCACCCAG	136

## Data Availability

Data available on request.

## Abbreviations

BCRP—breast cancer resistance protein, CD—cluster of differentiation, HDT—host-directed therapy, HIF—hypoxia-inducible factor, IL—interleukin, INFg—interferon gamma, *M.tb*—*Mycobacterium tuberculosis*, MRP1—multidrug resistance protein, PFA—perifocal area, P-gp—P-glycoprotein, SOCS—suppressor of cytokine signaling, STAT—Signal Transducers and Activators of Transcription, TB—tuberculosis, TGFb1—transforming growth factor-beta 1, TGM2—transglutaminase type 2, TNFa—tumor necrosis factor-alpha.
